# Supplementary material for: Conservation spillover effect of UNESCO World Heritage Sites into surrounding landscapes
Source: PeerJ. 2023 Oct 10;11:e15858. doi: 10.7717/peerj.15858 (PMC10573298; doi:10.7717/peerj.15858)
Supplement: Supplemental Information 1 [file peerj-11-15858-s001.docx]

**Table for Conservation spillover effect of UNESCO World Heritage Sites into surrounding landscapes**

Table 1. Dataset variable, resolution, and source information.

| Indicator | Variables | Resolution | Source |
| --- | --- | --- | --- |
| Human Footprint (HF) | Human Influence Index (HII) which includes human population pressure, land use and infrastructure, and human access variables. The grids are normalized by biome and realm. | 30 arc-second grid cell | Wildlife Conservation Society - WCS, and Center for International Earth Science Information Network - CIESIN - Columbia University. 2005. Last of the Wild Project, Version 2, 2005 (LWP-2): Global Human Footprint Dataset (Geographic). Palisades, New York: NASA Socioeconomic Data and Applications Center (SEDAC).<https://doi.org/10.7927/H4M61H5F>. Accessed 21 October 2021. |
| Forest Landscape Integrity Index (FLII) | Forest extent, observed and inferred human pressures, and forest connectivity loss. | 300 m | Grantham, H.S., Duncan, A., Evans, T.D. et al. (2020). Anthropogenic modification of forests means only 40% of remaining forests have high ecosystem integrity. Nat Commun 11, 5978. https://doi.org/10.1038/s41467-020-19493-3 |
| Global Landcover Data (LULC) | Landcover data. | 300 m | ESA GlobCover 2009 Project. GlobCover. due ESA. http://due.esrin.esa.int/page_globcover.php |
| World Heritage Site (WHS) | Point locations of all WHS. | N/A | UNESCO. (2022). *World Heritage Interactive Map.* UNESCO World Heritage Convention. https://whc.unesco.org/en/interactive-map/ |
| World Database on Protected Areas (WDPA) | All listed marine and terrestrial protected areas. The list is updated monthly. | N/A | UNEP-WCMC, IUCN (2022). Protected Planet: The World Database on Protected Areas (WDPA). https://www.protectedplanet.net/en. |
