# Supplementary material for: Conservation spillover effect of UNESCO World Heritage Sites into surrounding landscapes
Source: PeerJ. 2023 Oct 10;11:e15858. doi: 10.7717/peerj.15858 (PMC10573298; doi:10.7717/peerj.15858)
Supplement: Supplemental Information 2 — Description of each IUCN category and the count and percent of these categories for the selected World Heritage study sites. [file peerj-11-15858-s002.docx]

**Table for Conservation spillover effect of UNESCO World Heritage Sites into surrounding landscapes**

Table 2. Description of each IUCN category and the count and percent of these categories for the selected World Heritage study sites.

| IUCN Category | Definition | Count | Percentage (%) |
| --- | --- | --- | --- |
| Ia | Strict nature reserve | 7 | 1.83 |
| Ib | Wilderness area | 6 | 1.57 |
| II | National park | 74 | 19.37 |
| III | Natural monument or feature | 3 | 0.79 |
| IV | Habitat or species management area | 17 | 4.45 |
| V | Protected landscape or seascape | 43 | 11.26 |
| VI | Protected area with sustainable use of natural resources | 23 | 6.02 |
| Not applicable |  | 110 | 28.80 |
| Not Assigned |  | 15 | 3.93 |
| Not Reported |  | 84 | 21.99 |
